# Effectiveness of Disease-Modifying Therapies in Patients With Late-Onset Relapsing-Remitting Multiple Sclerosis

**DOI:** 10.1212/WNL.0000000000213967

**Published:** 2025-08-07

**Authors:** Sarmad Al-Araji, Marcello Moccia, Ashwani Jha, Le Zhang, Arman Eshaghi, Baris Kanber, Alessia Bianchi, Charmaine Yam, Omar Abdel-Mannan, Olivia Goodkin, Giuseppe Pontillo, Weaam Hamed, Suraya Mohamud, Wallace J. Brownlee, Declan T. Chard, Jeremy Chataway, Karen Chung, Floriana De Angelis, Ahmed Hammam, Yael Hacohen, Zhaleh Khaleeli, Siobhan M. Leary, Ferran Prados, Josephine Swanton, Alan J. Thompson, Sachid Anand Trip, Heather Wilson, Sarah Wright, Parashkev Nachev, Frederik Barkhof, Ahmed T. Toosy, Olga Ciccarelli

**Affiliations:** 1 Queen Square MS Centre, Department of Neuroinflammation, UCL Queen Square Institute of Neurology, London, United Kingdom;; 2 Department of Neurosciences, University Hospitals Coventry and Warwickshire, United Kingdom;; 3 Department of Molecular Medicine and Medical Biotechnology, Federico II University of Naples, Italy;; 4 National Institute for Health and Care Research (NIHR), University College London Hospitals (UCLH) Biomedical Research Centre, United Kingdom;; 5 High-Dimensional Neurology Group, Department of Brain Repair and Rehabilitation, UCL Queen Square Institute of Neurology, Faculty of Brain Sciences, University College London, United Kingdom;; 6 Centre for Medical Image Computing, Department of Medical Physics and Biomedical Engineering, University College London, United Kingdom;; 7 Cleveland Clinic London, Neurosciences Institute, United Kingdom;; 8 National Hospital for Neurology and Neurosurgery, London, United Kingdom;; 9 Department of Neurology, Great Ormond Street Hospital for Children, London, United Kingdom;; 10 eHealth Center, Universitat Oberta de Catalunya, Barcelona, Spain; and; 11 Department of Radiology and Nuclear Medicine, VU University Medical Centre, Amsterdam, the Netherlands.

## Abstract

**Background and Objectives:**

The benefit of disease-modifying therapies (DMTs) in relapsing-remitting multiple sclerosis (RRMS) is believed to decrease with age. We aimed to compare disease outcomes with DMTs between patients with late-onset RRMS (LO-RRMS) and adult-onset RRMS (AO-RRMS).

**Methods:**

This was a single-center, longitudinal, prospective analysis of patients who fulfilled the following criteria: (1) a diagnosis of RRMS and (2) initiation of a new DMT (dimethyl fumarate, fingolimod, glatiramer acetate, natalizumab, and ocrelizumab) within 3 months. Patients were followed up at years 1 and 2. We compared treatment outcomes (relapses, radiologic activity, disability progression, and no evidence of disease activity [NEDA]) between LO-RRMS (defined as age at onset of first symptom >45 years) and AO-RRMS (≥18 years and 45 years) using Poisson, logistic, and Cox regression models while adjusting for baseline variables. The analyses were repeated with an age cutoff of 50 years.

**Results:**

We studied 1,494 patients with AO-RRMS (mean age at onset: 29.6 years, 71% female) and 150 patients with LO-RRMS (50.2 years, 73% female) at treatment initiation. At DMT commencement, patients with LO-RRMS had shorter disease duration, higher Expanded Disability Status Scale (EDSS), more comorbidities, and were more likely to be treatment naive than patients with AO-RRMS. However, when adjusted, there were no differences in the probability of relapses (Coeff = −0.07; 95% CI −0.19 to 0.04, *p* = 0.24), EDSS progression (odds ratio [OR] 1.43, 95% CI 0.69–2.93, *p* = 0.33), MRI activity (OR 0.77; 95% CI 0.21–2.83, *p* = 0.70), and losing NEDA status (hazard ratio [HR] 0.93; 95% CI 0.73–1.18, *p* = 0.58) at year 1. Similar results were observed at year 2 in relapses (Coeff = −0.04; 95% CI −0.19 to 0.11, *p* = 0.60), EDSS progression (OR 1.33; 95% CI 0.69–2.93, *p* = 0.33), MRI activity (OR 1.30; 95% CI 0.38–4.38, *p* = 0.67), and loss of NEDA status (HR 0.99; 95% CI 0.77–1.26, *p* = 0.96). Similar results were observed using an age cutoff of 50 years. The percentages of patients who stopped DMTs because of side effects were similar between AO-RRMS and LO-RRMS.

**Discussion:**

Treatment outcomes over 2 years were similar between LO-RRMS and AO-RRMS. This indicates that care should be taken not to bias treatment decisions due to older age at onset of MS when patients demonstrate evidence of inflammatory activity. Limitations are the observational design, the single-center setting, and a relatively small LO-RRMS group.

**Classification of Evidence:**

This study provides Class III evidence that patients with LO-RRMS have comparable outcomes with DMTs as patients with AO-RRMS over a 2-year period; this rating is because of baseline imbalances between treatment groups and a nonmasked outcome assessment.

## Introduction

Multiple sclerosis (MS) is a chronic inflammatory demyelinating condition with major effect on physical and cognition domains, and employment. People with relapsing-remitting MS (RRMS) typically present with their first demyelinating event during their 20s and 30s.^1^ However, increasing incidence of MS onset of age older than 40–45 years has been observed in different populations over the past 20 years.^2^ More recently, a change in the distribution of age at onset has been described, with 2 peaks at around 30 years and 40–45 years of age.^3^ Overall, the MS population is aging, and the highest prevalence of MS was observed in the 45- to 64-year age group.^4^ Thus, studies on clinical care of RRMS patients with age at onset older than 45 years are highly relevant.

Chronologic age is a major factor in determining the clinical course of MS.^5,6^ RRMS patients with an older age at onset were more likely to exhibit disability progression and less likely to relapse compared with younger age at onset, independent of disease duration.^7,8^ The relapse rate has been observed to decrease with age, suggesting that disease-modifying therapies (DMTs) have the greatest efficacy in patients with onset younger than 40 years old.^9^ Delaying any DMT, even for a few years, lowers cumulative efficacy, and importantly, in the fourth decade of life, the efficacies of all DMTs overlap and become more difficult to predict the therapeutic effect of DMTs.^10,11^ However, the mean age of enrolment in clinical trials is usually younger than 50 years, and previous meta-analyses may have been underpowered to study the effect of age on DMT efficacy in individuals with an older age at onset.

Older age is also related to higher comorbidity risk, which may complicate the safety profile of DMTs, with increasing risk of infection with age.^12-14^ Overall, it has been suggested that older age is not only associated with lower DMT efficacy but also with higher frequency of adverse events.^15^ This is why people with late-onset MS may represent a vulnerable group, who are less frequently exposed to high-efficacy DMTs, and have a higher risk of disability progression than younger patients.^16^ Aging is associated with lower inflammatory activity, more widespread neurodegeneration, and reduced neuroplastic recovery mechanisms.^17^

The aim of this study was to compare treatment outcomes (number of relapses, disability worsening, MRI activity, and no evidence of disease activity [NEDA] status) at 1 and 2 years after the first DMT initiation between late-onset patients (LO-RRMS, who had their first relapse symptom at age of 46 years or older) and adult-onset RRMS (AO-RRMS, with age at onset between 18 and 45 years, inclusive of both ages), and determine whether treatment responses differed significantly between the 2 groups while adjusting for appropriate confounding variables.

## Methods

### Study Design and Participants

We combined the analysis of data from 2, real-world, single-center (Queen Square MS Centre, UCL) cohorts; both cohorts fulfilled the following inclusion criteria: (1) a diagnosis of RRMS according to the 2017 McDonald criteria^18^ and (2) initiation of any new DMT within 3 months. These 2 cohorts were (1) a cohort of all RRMS patients (both treatment naive and nonnaive) who commenced dimethyl fumarate, fingolimod, glatiramer acetate, and natalizumab, between 2002 and 2021; clinical and radiologic data were obtained by reviewing hospital electronic health records, which were collected prospectively as part of National Health Service (NHS) care. (2) A prospectively collected cohort of patients with consecutive RRMS who initiated ocrelizumab between 2019 and 2021 were assessed at the initiation of ocrelizumab and then at least annually, as part of the POINT-MS (Predicting Optimal INdividualised Treatment response in MS) study.

Patients were defined as LO-RRMS if their first clinical demyelinating event occurred older than 45 years of age and AO-RRMS if their first MS relapse event onset was between 18 and 45 years of age. The cutoff of 45 years was chosen because of the recently observed bimodal peak of the distribution of age at onset of MS, with the second peak being at about 40–45 years in Norway and 45–54 in Italy.^3,19^ There is currently no similar data available for the population in the United Kingdom.

For both cohorts, we collected data at study entry (within 3 months of DMT initiation), and at year 1 and year 2 after DMT initiation (±3 months). Expanded Disability Status Scale (EDSS) scores were collected for the first cohort from clinical notes at baseline, year 1, and year 2, either as documented or imputed from the described detailed neurologic examination and ambulation status. We included patients only once, which means that they did not contribute to the data set when they switched to a different DMT. Patients who discontinued their DMT before years 1 and 2 due to disease activity still contributed to NEDA assessment outcomes. Patients who stopped treatment because of other reasons, such as side effects, family planning, or relocation to a different MS service, were excluded from the analysis at year 1, if they stopped DMTs within the first 6 months, and at year 2, if they stopped DMTs within 18 months.

The following variables were collected at baseline (i.e., treatment initiation): age, sex (as recorded on the health records), ethnicity (self-identified), any comorbidity (due to the low number of observations, the variable used was the presence or absence of at least 1 comorbidity), age at MS relapse onset (used to calculate disease duration), number of relapses in the previous 12 months, EDSS, and brain MRI activity, defined as number of new and enlarging T2 and gadolinium-enhancing lesions (also referred to as “active lesions”) compared with the previous scans up to 24 months prior, as described in the neuroradiology reports. The demographics and baseline clinical information had minimal missing data and required no specific adjustment.

The outcomes collected at years 1 and 2 were: (1) total number of relapses (confirmed by the clinicians who reviewed the patients in clinic) from baseline to year 1 and from year 1 to year 2; (2) EDSS progression, defined as an increase of either ≥0.5 points for patients with a baseline EDSS score ≥5.5, ≥1.0 point for those with a baseline EDSS score from 1.0 to 5.0, and ≥1.5 points for those with a baseline EDSS score of 0; and (3) number of active MRI lesions seen on brain MRI from baseline to year 1 and from year 1 to year 2. In some instances where multiple MRI brain scans were performed within a 12-month period, the total number of active lesions was added up. (4) NEDA defined as the absence of relapses, EDSS progression, and active MRI lesions; this was calculated at year 1, year 2, and between years 1 and 2.

### Statistical Analysis

Cross-sectional comparisons of clinical, demographic, and radiologic characteristics between AO-RRMS vs LO-RRMS populations at baseline were performed using the *t* test, χ^2^ test or Fisher exact test, as appropriate.

We used the Mann-Whitney *U* test for dependent samples to investigate changes in new MRI lesions and relapses between baseline and year 1 and between baseline and year 2 within each patient group. Poisson regression was used to model the number of relapses at 1- and 2-year assessments between AO-RRMS and LO-RRMS. Logistic regression was used to test the difference in the probability of EDSS progression and the probability of brain MRI activity between the 2 groups at 1- and 2-year assessments; logistic regression was also used to test the differences in DMT discontinuation (any discontinuation, discontinuation due to side effects, discontinuation due to disease activity, and discontinuation due to other reasons) between groups. Cox regression was used to predict the risk of losing NEDA between AO-RRMS and LO-RRMS. All the models that investigated the differences between AO-RRMS and LO-RRMS were first run unadjusted to explore rates of different outcomes, and then adjusted for age, sex, ethnicity, comorbidities, disease duration, relapses in the previous year, baseline EDSS, active MRI lesions at baseline, number of previous DMTs, and type of DMT commenced, as covariates. We rerun the analysis without age as covariate. We also repeated all the analysis using an age cutoff of 50 years. We also evaluated an interaction term between group (AO-RRMS and LO-RRMS) and DMTs to test whether the outcome of interest differed between LO-RRMS and AO-RRMS depending on the DMT allocation. Finally, in a sensitivity analysis, we estimated adjusted and unadjusted statistical models for treatment naive patients only. For all models, the AO-RRMS categorical subgroup was used as reference.

Results are presented as estimated coefficients, odds ratios (ORs), hazard ratios (HRs), 95% CIs, and *p* values, as appropriate. Statistical analyses were performed using Stata 15.1.

Considering the observational design of this study with the inclusion of 1,494 patients with AO-RRMS and 150 patients with LO-RRMS, we calculated a power to detect 5.5% difference (in any outcome of interest) between groups, using regression models, with 5% alpha and 90% power.

### Standard Protocol Approvals, Registrations, and Patient Consents

Written consent was obtained from participants in the second cohort according to the protocol which was approved by the local Ethics Committee (19/WA/0157). In the first cohort, participants' consent was waived (23/WS/0008) (data available on the hospital electronic health records could be collected to develop precision treatment strategies in MS).

### Data Availability

Tabulated, anonymized data may be shared after appropriate approval is sought.

## Results

### Participants

**Figure 1:**
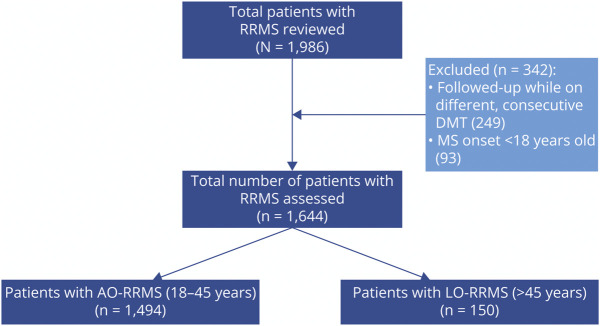
Flowchart of Patients With RRMS Included in Our Analysis AORRMS = adult-onset RRMS; DMT = disease-modifying therapy; LORRMS = late-onset RRMS; RRMS = relapsing-remitting multiple sclerosis.

A total of 1,494 patients with AO-RRMS (mean age at clinical onset: 29.6 years [SD = 7.0]) and 150 patients with LO-RRMS (mean age at clinical onset: 50.2 years [SD = 5.3]) were included at treatment initiation (Figure 1). Patients were included only once, that is, they did not contribute to the data set when they switched to a different, consecutive DMT. The proportion of patients with LO-RRMS was similar between the 2 cohorts (LO-RRMS represented 11% of the patients in the first cohort and 10% of those in the second cohort). Compared with AO-RRMS, patients with LO-RRMS had shorter disease duration, higher EDSS, were more often treatment naive and had at least 1 comorbidity at study entry (Table 1). The list of comorbidities which were encountered is given in eTable 1. Regarding type of DMTs, the same proportion of patients in each group were treated with ocrelizumab, but LO-RRMS was less often treated with natalizumab and fingolimod than AO-RRMS (Table 1). There were no significant differences between sex, ethnicity, number of relapses in the previous 12 months, and number of active MRI brain lesions at baseline (Table 1).

**Table 1:** Demographic and Clinical Characteristics at Baseline (or Treatment Initiation) for Both AO-RRMS (18–45 Years) and LO-RRMS (>45 Years)

	AO-RRMS (18–45 y) (n = 1,494)	LO-RRMS (>45 y) (n = 150)	*p* Value
Baseline, at DMT initiation			
Age at onset of their first symptom, y, mean ± SD	29.6 ± 7.0	50.2 ± 5.3	
18–25	445		
26–35	730		
36–45	319		
46–55		129	
56–65		18	
≥66		3	
Age at study entry (i.e., initiation of DMT), y, mean ± SD	38.0 ± 8.9	54.9 ± 6.9	
Sex, female	1,060 (71)	110 (73)	0.316
Disease duration, y, mean ± SD	8.4 ± 7.0	4.9 ± 4.5	<0.01^a^
Number of relapses in the previous year, mean ± SD	1.1 ± 0.9	1.0 ± 0.8	0.119
EDSS, median (range)	2.0 (0–8.0)	2.5 (0–7.0)	0.030^a^
Number (and proportion) of patients with at least 1 comorbidity	833 (56)	101 (67)	0.010^a^
Number of new T2 and enlarging lesions (compared with the previous scan within the previous 24 mo) and Gd+ lesions on brain MRI	1.2 ± 1.9	0.8 ± 1.5	0.104
Ethnicity (self-identified)			0.312
White	1,105 (74)	118 (79.0)	
Asian	146 (10)	11 (7)	
Black	78 (5)	8 (5)	
Mixed, other or unknown	165 (11)	13 (9)	
Number (and proportion) of patients who were treatment naive	684 (46)	99 (66)	<0.01^a^
Type of DMT			0.020^a^
Dimethyl fumarate	510 (34)	69 (46)	
Fingolimod	267 (18)	15 (10)	
Glatiramer acetate	380 (25)	39 (26)	
Natalizumab	151 (10)	7 (5)	
Ocrelizumab	186 (13)	20 (13)	

Abbreviations: AORRMS = adult-onset RRMS; DMT = disease-modifying therapy; EDSS = Expanded Disability Status Scale; Gd+ = gadolinium enhancing; LORRMS = late-onset RRMS; NEDA = no evidence of disease activity; RRMS = relapsing-remitting multiple sclerosis.

*p* Values are shown from *t* test, χ^2^ test, or Fisher exact test, as appropriate, comparing AO-RRMS and LO-RRMS.

a Statistically significant.

### Differences in Outcomes Between LO-RRMS and AO-RRMS at 1 and 2 Years

**Figure 2:**
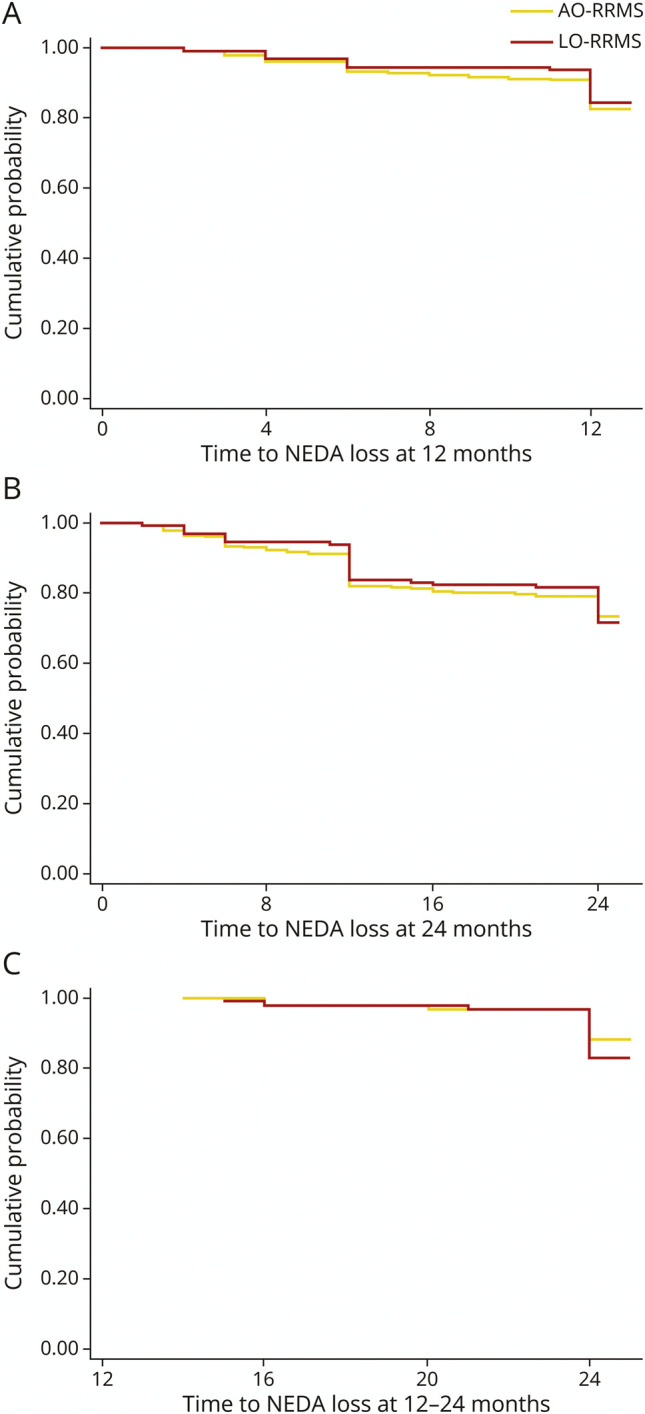
Time to NEDA Loss for AO-RRMS and LO-RRMS Kaplan-Meier estimates of the rate of losing NEDA at year 1 (A), year 2 (B), and between years 1 and 2 (C) for AO-RRMS (in yellow) and LO-RRMS (in red). AORRMS = adult-onset RRMS; LORRMS = late-onset RRMS; NEDA = no evidence of disease activity; RRMS = relapsing-remitting multiple sclerosis.

Clinical outcomes at year 1 and year 2 for each group are given in Table 2. The raw data showed that the AO-RRMS group had more relapses and MRI activity than LO-RRMS at each time point, but the reductions in number of new lesions and relapses from baseline to year 1 and from baseline to year 2 were significant within each group (all *p* < 0.01). There were no significant differences in the probability of relapses, EDSS progression, new MRI activity, and NEDA at year 1, year 2, and between year 1 and year 2, between AO-RRMS and LO-RRMS (mostly driven by EDSS progression in both groups; Table 2), when regression models were adjusted for age, sex, disease duration, ethnicity, comorbidities, relapses in the previous year, baseline EDSS, number of active MRI lesions at baseline, number of previous DMTs, and type of DMT used (Table 3 and Figure 2). All the statistical models were repeated using an age cutoff of 50 years, and the differences in treatment outcomes between AO-RRMS (N = 1,512) and LO-RRMS (N = 113) remained nonsignificant (eTable 2). We repeated the analyses without age as a covariate, and the results did not change (eTables 3 and 4).

**Table 2:** Clinical Outcomes at 1 and 2 Years for Both AO-RRMS (18–45 Years) and LO-RRMS (>45 Years)

	AO-RRMS (18–45 y) (n = 1,494)	LO-RRMS (>45 y) (n = 150)
Year 1		
Number (and proportion) of patients eligible for NEDA assessment at 1 y^a^	1,447 (97)	141 (94)
Mean number of relapses between baseline and year 1	0.2 ± 0.5	0.1 ± 0.4
Number (and proportion) of patients with relapses	191 (13)	14 (10)
EDSS, median (range)	2.0 (0–8.5)	2.5 (0–7.0)
Number (and proportion) of patients with EDSS progression	61 (4)	9 (6)
Mean number of new and enlarging T2 lesions and Gd+ lesions on brain MRI at year 1 when compared with baseline	0.3 ± 1.0	0.1 ± 0.4
Number (and proportion) of patients with new and enlarging T2 lesions and Gd+ lesions on brain MRI at year 1 when compared with baseline	107 (7)	4 (3)
Number (and proportion) of patients who showed NEDA at year 1 vs baseline	1,182 (82)	118 (84)
Year 2		
Number (and proportion) of patients eligible for NEDA assessment at 2 y^a^	1,218 (82)	118 (79)
Mean number of relapses between year 1 and year 2	0.2 ± 0.5	0.1 ± 0.6
Number (and proportion) of patients with relapses	165 (14)	13 (11)
EDSS, median (range)	2.5 (0–8.5)	2.5 (0–7.5)
Number (and proportion) of patients with EDSS progression	80 (7)	16 (14)
Mean number of new and enlarging T2 lesions and Gd+ lesions on brain MRI at year 2 when compared with year 1	0.2 ± 0.8	0.1 ± 0.4
Number (and proportion) of patients with new and enlarging T2 lesions and Gd+ lesions on brain MRI at year 2 when compared with year 1	90 (7)	6 (5)
Number (and proportion) of patients who showed NEDA at year 2 vs baseline	811 (67)	80 (68)
Number (and proportion) of patients who showed NEDA between years 1 and 2	909 (75)	88 (75)

Abbreviations: AORRMS = adult-onset RRMS; EDSS = Expanded Disability Status Scale; LORRMS = late-onset RRMS; NEDA = no evidence of disease activity; RRMS = relapsing-remitting multiple sclerosis.

a If patients switched to a different DMT during the study period because of disease activity or disease progression they contributed with their first DMT and were not excluded. Note that patients may have experienced more than 1 NEDA component over time, and therefore, the number of patients achieving NEDA may be different from that obtained when adding the individual NEDA components.

**Table 3:** Results of Regression Analyses That Compared the Risk of Relapses, EDSS Progression, New/Gd+ Lesions MRI, and NEDA at Year 1 and Year 2 Between AO-RRMS (Which Is the Reference Group) and LO-RRMS

	Adjusted			
		95% CI		*p* Value
		Upper	Lower	
Year 1				
Number of relapses	Coeff −0.07	−0.19	0.04	0.236
EDSS progression	OR 1.43	0.69	2.93	0.328
New and enlarging T2 lesions and Gd+ lesions on brain MRI	OR 0.77	0.21	2.83	0.699
NEDA at year 1	HR 0.93	0.73	1.18	0.575
Year 2				
Number of relapses	Coeff −0.04	−0.19	0.11	0.601
EDSS progression	OR 1.33	0.77	2.27	0.296
New and enlarging T2 lesions and Gd+ MRI lesions on brain MRI	OR 1.30	0.38	4.38	0.672
NEDA at year 2	HR 0.99	0.77	1.26	0.959
NEDA between years 1 and 2	HR 1.07	0.80	1.42	0.642

Abbreviations: AORRMS = adult-onset RRMS; DMT = disease-modifying therapy; EDSS = Expanded Disability Status Scale; Gd+ = gadolinium enhancing; LORRMS = late-onset RRMS; NEDA = no evidence of disease activity; RRMS = relapsing-remitting multiple sclerosis.

Results of Poisson regression (number of relapses), logistic regression (probability of EDSS progression, probability of new T2 and Gd+ MRI lesions), and Cox regression models (probability of losing NEDA), comparing AO-RRMS and LO-RRMS. First, we ran unadjusted models, and then, we included age, sex, ethnicity, comorbidities, disease duration, relapses in the previous year, baseline EDSS, new T2 and/or Gd+ lesions at baseline, number of previous DMTs, and DMT allocation, as covariates (adjusted models). The AO-RRMS subgroup was used as reference in the statistical models, so results directly refer to LO-RRMS. Note that when the same analyses were repeated without age as covariate, the results were substantially similar (see eTable 3).

The proportion of patients who discontinued therapy for reasons other than disease activity was similarly low in both groups (3% [N = 47] in the AO-RRMS group and 6% [N = 9] in the LO-RRMS group). Among these, the majority discontinued due to side effects (77% in the AO-RRMS group and 67% in the LO-RRMS group); these patients were therefore not eligible for NEDA assessment at years 1 and 2, after adjusting for all confounding variables (eTable 5).

Finally, when we repeated the main analysis that compared the outcomes at year 1 and year 2 between AO-RRMS and LO-RRMS with only treatment-naive patients, the findings remained materially unaltered (Table 4).

**Table 4:** Results of Regression Analyses That Compared the Outcomes at Year 1 and Year 2 Between AO-RRMS (Which Is the Reference Group) and LO-RRMS When Considering Treatment-Naive Patients

	Adjusted			
		95% CI		*p* Value
		Upper	Lower	
Year 1				
Number of relapses	Coeff 0.04	−0.09	0.19	0.515
EDSS progression	OR 1.77	0.64	4.89	0.267
New and enlarging T2 lesions and Gd+ lesions on brain MRI	OR 0.84	0.16	4.29	0.836
NEDA at year 1	HR 0.99	0.71	1.37	0.975
Year 2				
Number of relapses	Coeff −0.06	−0.26	0.13	0.529
EDSS progression	OR 1.92	0.93	3.95	0.076
New and enlarging T2 lesions and Gd+ lesions on brain MRI	OR 0.71	0.14	3.51	0.682
NEDA at year 2	HR 1.01	0.73	1.39	0.940
NEDA between years 1 and 2	HR 1.00	0.68	1.47	0.983

Abbreviations: AORRMS = adult-onset RRMS; DMT = disease-modifying therapy; EDSS = Expanded Disability Status Scale; Gd+ = gadolinium enhancing; LORRMS = late-onset RRMS; NEDA = no evidence of disease activity; RRMS = relapsing-remitting multiple sclerosis.

Results of Poisson regression (number of relapses), logistic regression (probability of EDSS progression, probability of new T2 and Gd+ MRI lesions), and Cox regression models (probability of NEDA), comparing treatment-naive AO-RRMS and LO-RRMS. First, we run unadjusted models, and then, we included age, sex, ethnicity, comorbidities, disease duration, relapses in the previous year, baseline EDSS, new T2 and Gd+ lesions at baseline, and DMT allocation, as covariates (adjusted models). The AO-RRMS subgroup was used as reference in the statistical models, so results directly refer to LO-RRMS. Note that when the same analyses were repeated without age as covariate, the results were substantially similar (see eTable 4).

When including the interaction term between RRMS groups (AO-RRMS and LO-RRMS) and DMTs to the previous statistical models, we observed no significant associations, suggesting that all outcomes were equally reached by AO-RRMS and LO-RRMS independently from DMTs, indicating a similar treatment response (eTable 6).

### Classification of Evidence

This study provides Class III evidence that patients with LO-RRMS have comparable outcomes with DMTs as patients with AO-RRMS over a 2-year period; this rating is because of baseline imbalances between treatment groups and a nonmasked outcome assessment.

## Discussion

Although patients with LO-RRMS initiated DMTs with higher disability and more comorbidities when compared with AO-RRMS, they showed similar clinical and MRI outcomes over 2 years after DMT initiation. Comparable results were observed when only treatment naive patients were considered. In addition, there were no differences between LO-RRMS and AO-RRMS in their response to specific DMTs and the proportion of patients who stopped because of side effects.

It has been suggested that the benefits of DMTs decrease with age.^8-10^ Some randomized controlled trials, which excluded patients older than 55 years, reported a reduced treatment effect in patients older than 40 years.^19^ However, we found that the apparent differences in treatment outcomes (number of relapses, disability progression, MRI activity, and NEDA status) between AO-RRMS and LO-RRMS became nonsignificant when adjusting for age, sex, disease duration, ethnicity, comorbidities, relapses in the previous year, baseline EDSS, baseline clinical MRI activity, number of previous DMTs, and type of DMT used, suggesting that the differences in the raw data between groups may have been influenced by other factors, although adjusting for additional variables may have reduced the statistical power of the test. The observed results did not substantially change when age was not included as a covariate. The raw data showed that the AO-RRMS group had more relapses and MRI activity than LO-RRMS at each time point, but the reductions in number of new lesions and relapses from baseline to follow-ups were significant within each patient group, indicating that the lack of difference between groups was not due to the lack of events. Overall, our findings are in line with another trial, where the upper age limit for enrolment was 65 years, which demonstrated similar efficacy between patients older than 40 years and younger patients.^20^ Therefore, our findings clearly demonstrate that DMTs, including high-efficacy therapies, are as beneficial in LO-RRMS as in AO-RRMS over 2 years, thereby suggesting that age at onset alone should not prevent patients with older age at onset to initiate a DMT.

Although previous studies have suggested that age is an important risk factor for disability accrual in RRMS,^11,21^ we did not find any difference in the probability of EDSS worsening between the AO-RRMS and LO-RRMS groups over 2 years after DMT initiation. When we performed a sensitivity analysis with an age cutoff of 50 years, instead of 45, no differences in treatment outcomes between AO-RRMS and LO-RRMS were seen, but the group over 50 years was smaller in size (n = 113), potentially reducing power. It is important that the number of patients 66 years or older of age was very low (n = 3), suggesting that caution should be taken when generalizing these results to the very old. A meta-analysis of 38 randomized clinical trials found that, after the age of 53 years, there is no benefit from DMTs in disability progression^11^; however, this meta-analysis was based on clinical trials (before 2017) that excluded patients of age older than 50 years, hence was potentially underpowered for this older age group. A recent multicenter, observational, retrospective Italian cohort study found that relapses predicted 12-month confirmed disability worsening in LO-RRMS, and DMTs reduced the risk of reaching a sustained EDSS score of 4.0 in patients with LO-RRMS.^22^ In the future, it may be interesting to address the differences in progression independent of relapses and MRI activity (PIRMA) associated with treatment response, but the assessment of PIRMA as outcome of treatment is challenging and requires special methodologic considerations.^23^

A large discussion on DMT in older patients also focuses on risks. Patients with LO-RRMS showed more comorbidities at treatment initiation than AO-RRMS, suggesting that the treating neurologists were prescribing DMTs for older patients who may have appeared to be “less healthy” than AO-RRMS. It is important that the risk of selection bias of this real-world population may have been limited by the fact that patients are treated as per national guidelines, and DMTs were discussed at disease-modifying therapy multidisciplinary team meetings. A strength of this study is assessing adverse effect comparisons between the 2 groups. We found no significant difference in the proportion of patients who stopped DMTs due to side effects. However, we did not have the data to look at the differences in the nature or severity of adverse events between older and younger patients. Long-term data on the DMT tolerability and safety are needed, and a recent study has reported that these can be obtained from administrative health data.^24^ A recent real-world observational study found that in patients 50 years and older with nonactive MS, the risk of relapse was significantly higher over 2 years after discontinuation of high-efficacy therapy (including natalizumab and fingolimod) compared with the continuation group,^25^ suggesting that relaying DMTs for active, although older, patients should be considered.

At the time of treatment initiation, patients with LO-RRMS had higher disability (EDSS 2.5 vs 2.0), compared with patients with AO-RRMS, although this was overall mild disability, thereby indicating that there was scope to intervene to try to prevent disability accumulation. Considering the shorter disease duration between onset and initiation of DMT in the LO-RRMS group than AO-RRMS, it is unlikely that the higher disability was due to a delay in diagnosis or in the time to DMT initiation.^26,27^ Therefore, it is more likely that this was due to a more rapid disability accrual in late-onset than adult-onset patients. In our center, considering any DMT, either as first therapy or after switching, the proportions of patients who started ocrelizumab, dimethyl fumarate, and glatiramer acetate were similar between AO-RRMS and LO-RRMS. However, the proportions of patients on natalizumab were higher in the AO-RRMS group than in the LO-RRMS, which could have been a potential bias toward lower disease activity in the patients with AO-RRMS, which was not observed; additionally, the type of DMT used and the number of previous DMTs used were both adjusted for in our analysis. Also in other centers, patients with LO-RRMS have been reported to be less likely to receive high-efficacy DMTs than AO-RRMS.^16,28^ Future studies may focus on investigating treatment outcomes when patients are stratified into groups on the basis of their disability.

Since our study is observational and nonrandomized, we acknowledge several limitations. Different DMTs were offered at different periods over the past 2 decades, depending on treatment availability, which could represent a potential confounder over time; ocrelizumab became available in the United Kingdom in 2019, and the evidence that early initiation of high-efficacy therapy is associated with lower disability after 6–10 years compared with delayed commencement in the disease course was published in 2020.^29^ Therefore, to account for the use of anti-CD20 in most recent years, we have merged the 2 cohorts to consider the most recent introduction of ocrelizumab. The first cohort was based on the use of hospital records which are recorded prospectively, while the second cohort was based on patients' active enrolment; however, the proportion of patients with LO-RRMS was similar in both cohorts, and patients were consecutive.

Our results have shown no significant difference between the outcomes of the 2 groups despite most variables indicating numerically higher inflammatory activity in the AO-RRMS group at each time point. Although the AO-RRMS sample size was large, the sample size of the LO-RRMS was not. However, we have collected one of the largest LO-RRMS groups reported with clinical and radiologic data and repeated the analysis with an age cutoff of 50 years. Although our first set of results included age as a covariate, we subsequently reran the analyses after dropping age and the results remained substantially similar.

A possible limitation is that the proportions of patients with EDSS progression over the 2-year follow-up were 7% in AO-RRMS and 14% in the LO-RRMS, which are higher than those observed in recent randomized clinical trials^30^; however, EDSS worsening was not confirmed at 3 or 6 months, and this applied equally to the patients in both cohorts.

The length of follow-up of patients on treatment is of 2 years, which is short, although the same as most randomized clinical trials; longer follow-up studies on treatment outcomes, including safety, are needed. In addition, we did not calculate the percentage of patients with LO-RRMS and AO-RRMS who started DMTs out of all patients seen at our MS center, but the proportion of LO-RRMS is expected to be lower than that of AO-RRMS (RRMS patients with older age at symptom onset was found to be about 10% in the UK MS Register and 12% in the Swedish Register).^8,16^

Finally, we only included MRI brain lesions, and future work will aim to include spinal cord MRI scans for complete evaluation. On the other hand, the strengths of our work include the representation of daily clinical MS practice and the homogeneity in treatment decisions, because of NHS England treatment algorithm and multidisciplinary meetings that respectively guide and approve the choice of DMT; in addition, a large number of patients are followed at our center, thus reducing variability of data collection and access to DMT.

In conclusion, patients with LO-RRMS initiated DMTs with higher disability and comorbidities, but treatment outcomes were comparable with AO-RRMS. Our study suggests that age alone should not prevent patients being considered for DMTs because, if eligible with evidence of inflammatory disease activity, there is a similar probability of responding over 2-year follow-up.

## Glossary

AORRMS: adult-onset RRMS
DMT: disease-modifying therapy
EDSS: Expanded Disability Status Scale
HR: hazard ratio
LORRMS: late-onset RRMS
MS: multiple sclerosis
NEDA: no evidence of disease activity
NHS: National Health Service
OR: odds ratio
PIRMA: progression independent of relapses and MRI activity
RRMS: relapsing-remitting MS
